# Adipokines: A gear shift in puberty

**DOI:** 10.1111/obr.13005

**Published:** 2020-01-30

**Authors:** Desirée Nieuwenhuis, Natàlia Pujol‐Gualdo, Ilse A.C. Arnoldussen, Amanda J. Kiliaan

**Affiliations:** ^1^ Department of Anatomy Radboud university medical center, Donders Institute for Brain, Cognition and Behaviour, Preclinical Imaging Center PRIME Nijmegen The Netherlands

**Keywords:** adipokines, obesity, puberty

## Abstract

In this review, we discuss the role of adipokines in the onset of puberty in children with obesity during adrenarche and gonadarche and provide a clear and detailed overview of the biological processes of two major players, leptin and adiponectin. Adipokines, especially leptin and adiponectin, seem to induce an early onset of puberty in girls and boys with obesity by affecting the hypothalamic‐pituitary‐gonadal (HPG) axis. Moreover, adipokines and their receptors are expressed in the gonads, suggesting a role in sexual maturation and reproduction. All in all, adipokines may be a clue in understanding mechanisms underlying the onset of puberty in childhood obesity and puberty onset variability.

## 1 INTRODUCTION

The prevalence of obesity in adolescents and children is increasing in alarming rates.^1^, ^2^ Specifically, worldwide, 41 million children below the age of 5 years were overweight or were with obesity in 2016, and this number is expected to increase to 70 million in 2025.^3^ Childhood obesity is associated with various severe health complications, including increased risk of diabetes mellitus type 2, hypertension, heart diseases, and disturbances in sex hormone levels.

Obesity is defined by an excessive accumulation of white adipose tissue (WAT), and it is often indicated by a body mass index (BMI) above 30.^4^ Two main types of adipose tissue were described: WAT and brown adipose tissue (BAT), which differ in morphology and function.^5^, ^6^ BAT consists of adipocytes containing multiple lipid droplets and mitochondria and plays a role in thermogenesis. Adipocytes in WAT contain only a few mitochondria and a single lipid droplet.^5^, ^6^, ^7^ Adipose tissue has several functions including the storage of energy, thermogenesis, and the production and secretion of adipokines (hormones, cytokines, and peptides).^5^, ^7^, ^8^ Adipokines are involved in a number of physiological processes including blood pressure, metabolism, glucose, and vascular homeostasis and may play amongst others a key role in puberty onset.^8^, ^9^, ^10^

Puberty is known as a period through which the body changes physically, being a physiological process resulting in the maturation of children, i.e. they develop sexual characteristics and obtain reproductive functions.^9^, ^11^ Although many studies have shown associations between obesity and puberty,^2^, ^12^, ^13^, ^14^, ^15^, ^16^, ^17^, ^18^, ^19^, ^20^, ^21^, ^22^, ^23^ the biological mechanisms underlying obesity and puberty onset remain unclear. Hereafter, we review in detail the role of adipokines in the onset of puberty in childhood obesity.

## 2 PHYSIOLOGICAL PROCESSES IN THE INITIATION OF PUBERTY

**Figure 1:**
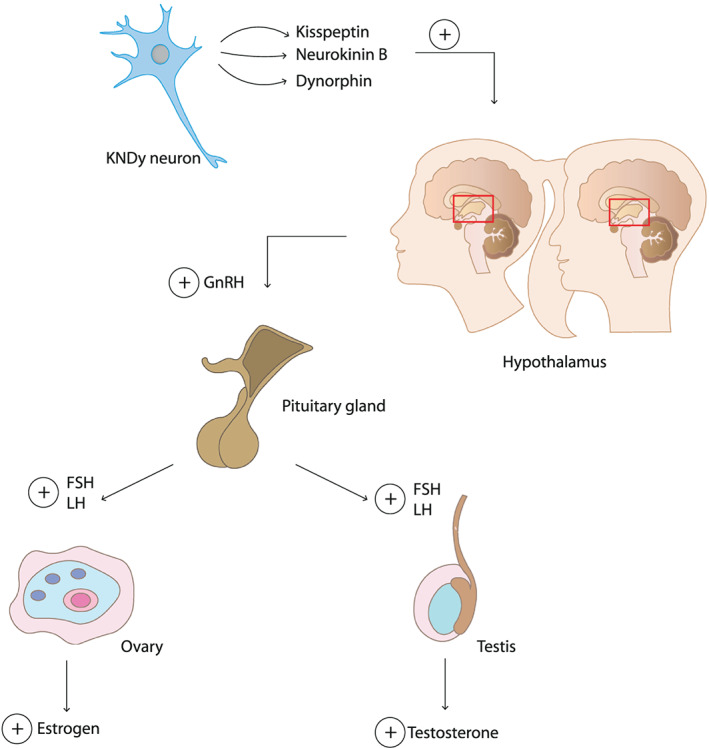
Hormonal regulation in the initiation of puberty in boys and girls. The secretion of kisspeptin, neurokinin B, and dynorphin from KNDy neurons initiate the release of gonadotropin releasing hormone (GnRH) from the hypothalamus. This activates the pituitary gland to produce and secrete luteinising hormone (LH) and follicle stimulating hormone (FSH), which in turn stimulate the gonads to produce estrogen and testosterone in girls and boys, respectively

Generally, two physiological processes, adrenarche and gonadarche, interact to regulate the onset of puberty.^11^, ^24^ During adrenarche, the adrenal cortex secretes steroid hormones (including androstenedione, dehydroepiandrosterone, dehydroepiandrosterone sulfate (DHEAS), androstenedione, and cortisol), insulin‐like growth factor, and growth hormone, which contribute to the pubertal growth spurt, body odor, skin oiliness, and skeletal maturation.^9^, ^24^, ^25^ Both adrenarche and gonadarche are involved in the development of pubic hair.^25^ During gonadarche (Figure 1), the hypothalamic‐pituitary‐gonadal (HPG) axis is activated,^2^, ^26^ and several hormones have been identified to participate in the activation of the HPG axis including kisspeptin, neurokinin B, dynorphin, leptin, and ghrelin.^2^, ^27^ Kisspeptin, neurokinin B, and dynorphin are released by specialized neurons, the KNDy neurons in the hypothalamus.^28^ Kisspeptin is a key regulator of the pulsatile secretion of gonadotropin releasing hormone (GnRH) from the hypothalamus.^29^, ^30^ In addition, neurokinin B stimulates, and dynorphin inhibits the release of kisspeptin, which implies that both coordinate a pulsatile release of kisspeptin.^31^ Subsequently, the activated HPG axis induces the pituitary gland to secrete luteinising hormone (LH) and follicle stimulating hormone (FSH). As a result, gametogenesis occurs, and the gonads will release sex hormones. Consequently, secondary sex characteristics develop including breast development in girls and an increased testicular volume in boys.^2^, ^26^, ^32^

The age at puberty onset varies greatly among individuals, which is possibly due to differences in levels of body fat, hypothalamic‐pituitary‐adrenal (HPA) axis activity, and genetic background.^33^ Recent genome‐wide association studies have provided important new insights on new genetic loci (e.g. melanocortin‐4 receptor, mitochondrial carrier 2, and mitogen‐activated protein kinase 13) and on several pathways that regulate the timing of puberty; however, it partly explains puberty timing variation.^34^ Thereby, defining the role of adipokines is of importance in elucidating the variability in puberty as the expression of adipokines is sex‐specific and is altered with body composition, adiposity, and during growth spurts. Moreover, adipokines and their receptors are expressed in gonads and several brain regions suggesting involvement in the onset of puberty and sexual maturation. Lastly, adipokines interfere in processes regulating timing and duration of puberty, for instance in the HPA and HPG axes which are both key players during adrenarche and gonadarche. Involvement of adipokines in the onset of puberty and specifically in individuals with obesity will be further reviewed in the next sections.^2^, ^24^

## 3 THE ONSET OF PUBERTY IN GIRLS

Puberty onset in girls is assessed using different markers, such as thelarche (breast development), menarche (the start of menstruation),^19^ and pubic hair development.^35^ The average age of girls at start of menarche is 12.4 years.^36^ However, this age differs between cultures and ethnicities, and since 1980, age at menarche is significantly decreasing.^36^, ^37^, ^38^, ^39^

### 3.1 Fat storage

For the initiation of puberty, the timing of stimulation and/or inhibition of different hormones is important, and additionally, a certain amount and distribution of body fat is needed in order to start menarche, which emphasizes the importance of body fat. From an evolutionary point of view, body fat increases in mammalian females during puberty onset, and it highlights the need to guarantee a healthy pregnancy, offspring, and maternal survival.^40^ An improper level of body fat, sex‐hormones, and neuroendocrine alterations can evolve in menstrual dysfunction, for instance, in women with severe obesity or in women with anorexia nervosa.^41^, ^42^, ^43^ Importantly, body fat distribution, particularly body fat localized predominantly on the gluteofemoral fat depots, is profoundly associated with start of menarche, more than amount of total body fat.^44^, ^45^, ^46^ Blood leptin levels are strongly related to gluteofemoral fat depots suggesting that leptin may convey information on body fat distribution to the hypothalamus during puberty.^45^

### 3.2 HPG axis

The HPG axis is activated by the release of kisspeptin resulting in the release of GnRH from the hypothalamus, and LH and FSH from the pituitary gland. In girls, FSH is involved in the development of the follicles in the ovaries, and it promotes the secretion of estrogen. LH stimulates the production of androgen hormones and induces ovulation (Figure 1).^9^, ^47^ The secretion of estrogen has an inhibitory effect on the release of kisspeptin and neurokinin B, and kisspeptin thereby inhibits the GnRH release from the hypothalamus.^48^ The expression pattern of GnRH is important for the regulation of the menstrual cycle. This roughly 28‐day‐cycle comprises several phases, including the follicular phase and luteal phase. During the follicular phase, increasing levels of FSH stimulate the maturation of follicles and the production of estrogen from the ovaries. This in turn inhibits the release of FSH from the pituitary gland. A high level of estrogen will induce the production of LH by the pituitary gland, resulting in ovulation. The matured follicle secretes progesterone thereby inhibiting the release of GnRH. When the corpus luteum is demolished, there is less inhibition of GnRH. As a consequence, the cycle will start again.^48^ This whole process, starting from the activated HPG axis, results in the development of the secondary sex characteristics in girls including thelarche and menarche.^9^, ^47^

### 3.3 Adipokines

According to results from studies reported in Table 1, girls with obesity enter puberty earlier compared with girls with normal weight.^13^, ^14^, ^16^, ^17^, ^18^, ^19^, ^20^, ^21^, ^22^, ^23^, ^49^, ^50^, ^51^ An explanation for the early onset of puberty in these girls might be found in the secretion of adipokines. For instance, leptin is positively associated with the amount of body fat. Generally, higher leptin concentrations inhibit the intake of food and increases energy expenditure.^9^, ^52^, ^53^, ^54^

**Table 1:** Summary of included studies

Authors	Year	Country	Study Design	Primary Outcome	Sex	Sample Size (n)	Age (y)	Data Collection
Lian et al^21^	2019	China	Cross‐sectional	Puberty starts earlier in Chinese Han girls with obesity compared with Chinese Han girls with normal weight.	Girls	2996	9‐19	2012 and 2013
Biro et al^12^	2018	USA	Longitudinal	Body mass index had a greater effect on age at menarche than did race and ethnicity.	Girls	946	6‐16	2004‐2014
Lazzeri et al^20^	2018	Italy	Cross‐sectional	Overweight during childhood shows a relation with the early onset of puberty in girls.	Girls	6535	11	2009/2010
					Girls	4259	15	2013/2014
Li et al^23^	2018	China	Longitudinal	For both, boys and girls, a higher BMI (ie, overweight and obese) is associated with earlier onset of puberty	Boys	695	5.8‐12.2	2014‐2017
					Girls	542		
Deng et al^22^	2017	China	Cross‐sectional	Increased BMI is associated with early timing spermarche and menarche.	Boys	1278258	9‐15	2005‐2012
					Girls			
Flom et al^15^	2017	USA	Prospective birth cohort	Overweight/obese status at the age of 7 ye was associated with increased risk of early menarche	Girls	788	From birth to menarche occurred	Pregnancies 1959‐1966
He et al^24^	2017	China	Cross‐sectional	Onset of puberty is not related to obesity in boys.	Boys	782	7‐17	
Holmgren et al^17^	2017	Sweden	Longitudinal	Higher BMI during childhood is associated with early puberty.	Boys	972		2008 and
					Girls	929		2009
Kelly et al^19^	2017	UK	Longitudinal prospective cohort	Higher BMI in girls is associated with the onset of menstruation at an earlier age.	Girls	5839	11	2000‐2002
Barcellos Gemelli et al^25^	2016	Brazil	Cross‐sectional	Excess weight is associated with early age of menarche.	Girls	727	10‐18	2014
Glass et al^16^	2016	USA	Longitudinal	In girls, but not in boys, greater adiposity is associated with the earlier onset of puberty.	Boys	123	11‐17	2003‐2009
					Girls	135		
Lee et al^26^	2016	USA	Cross‐sectional	Boys with overweight enter puberty earlier compared with boys with normal weight or obesity, while puberty starts later in boys with obesity compared with boys with normal weight and overweight.	Boys	3872	6‐16	2005‐2010
Cabrera et al^27^	2014	USA	Cross‐sectional	Thelarche occurred earlier than recently reported, while age of menarche remained unchanged.	Girls	610	3‐17.9	2007
Leonibus et al^14^	2013	Italy	Longitudinal	Obesity during childhood is related to the earlier onset of puberty.	Boys	71		2005‐2012
					Girls	84		
Currie et al^13^	2012	Europe, USA, Canada	Cross‐sectional	Overweight/obesity during childhood predicts the early onset of puberty in girls.	Girls	20410	11, 13, 15	2005‐2006
Herman‐Giddens et al^28^	2012	USA	Cross‐sectional	Observed mean ages of beginning genital and pubic hair growth and early testicular volumes were earlier than in past studies, depending on the characteristic and race/ethnicity.	Boys	4131	6‐16	2005‐2010
Sorensen et al^29^	2010	Denmark	Cross‐sectional/longitudinal	Puberty onset at earlier ages was associated with an increased BMI in boys.	Boys	1528	5.8‐19.9	1991‐1993/2006‐2008
Aksglaede et al^30^	2009	Denmark	Longitudinal	The higher BMI in boys and girls at 7 y of age, the earlier they enter puberty.	Boys	21 612		1930‐1969
					Girls	135 223		
Juul et al^31^	2007	Denmark	Retrospective cohort	Higher BMI is associated with early voice break.	Boys	463	11‐15	1990‐1999
Ribeiro et al^32^	2006	Portugal	Cross‐sectional	Early sexual maturation in boys and girls is associated with overweight.	Boys	382	10‐15	
					Girls	437		
Kaplowitz et al^18^	2001	USA	Cross‐sectional	The early onset of puberty in Caucasian girls is likely related to an increased BMI.	Girls	10 750	5‐12	1992‐1993

Abbreviation: BMI, body mass index.

Leptin may possibly play a role in adrenarche as its plasma level increases with higher levels of body fat and as it can modulate both the HPA and HPG axes.^33^ These axes are functionally integrated during adrenarche. In coherence, in children with obesity, the androgen DHEAS was positively associated with leptin levels.^55^ Nevertheless, another study showed that enhanced adrenal androgen secretion in girls with premature adrenarche was not explained by leptin or BMI levels.^55^ In addition, the adipokine adiponectin was negatively associated with androgen levels in girls^56^; however, it was not related to adrenarche in girls with Prader‐Willi syndrome.^57^ Interestingly, sex differences of adiponectin seem to develop during the progression of puberty.^56^ Thus, leptin and adiponectin might be able to influence adrenarche; however, both are not required factors.

**Figure 2:**
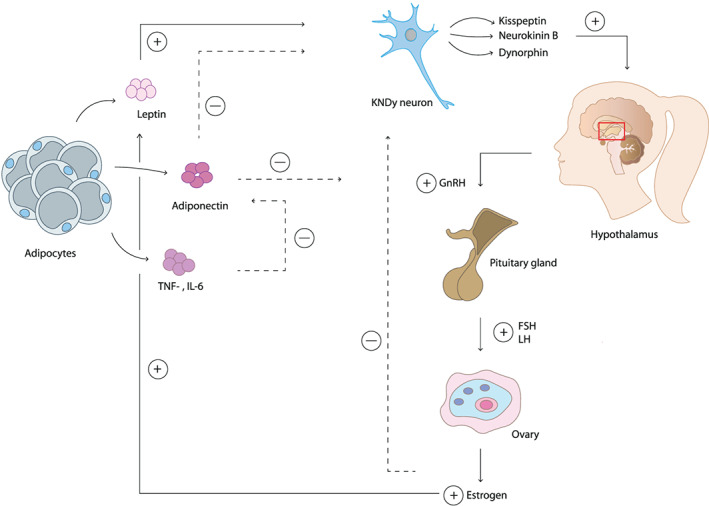
Adipokines affecting the initiation of puberty in girls. Leptin stimulates the release of kisspeptin in KNDy neurons, which activates the hypothalamus to produce gonadotropin releasing hormone (GnRH). In response to the release of GnRH, the pituitary gland secretes follicle stimulating hormone (FSH) and luteinising hormone (LH), which stimulates the ovaries to release estrogen resulting in the formation of secondary sex characteristics in girls. Estrogen stimulates the production of leptin. Adiponectin inhibits GnRH release resulting in reduced levels of GnRH and thereby a delayed onset of puberty. TNF‐α and IL‐6 inhibit the production of adiponectin and therefore stimulate the onset of puberty

In gonadarche, leptin can stimulate the secretion of kisspeptin, and subsequently activation of the HPG axis, which eventually increases the expression of estrogen and androstenedione in the ovaries (Figure 2).^58^ In return, estrogen stimulates the expression of the Ob gene in WAT, resulting in the synthesis and secretion of leptin.^59^ Thus, high levels of leptin promote onset of puberty in girls via secretion of kisspeptin, and estrogen stimulates leptin secretion additionally. Moreover, adiponectin can affect the HPG axis due to the expression of adiponectin receptors in the hypothalamus, pituitary gland, and gonads.^2^, ^60^ In detail, adiponectin is a regulator of puberty onset as it inhibits the secretion of kisspeptin and GnRH in the hypothalamus and the release of GH and LH in the pituitary gland, and thereby inhibiting the onset of puberty (Figure 2).^2^, ^60^, ^61^, ^62^ Individuals with obesity often have low levels of adiponectin.^52^, ^60^ Sitticharoon et al. showed that total adiponectin was significantly lower, whereas high molecular weight (HMW) adiponectin was significantly higher in girls with central precocious puberty (CPP).^63^ Moreover, total adiponectin had negative correlations with progression of puberty in girls (defined by Tanner stages), whereas HMW adiponectin had positive associations with LH levels and the progression of puberty in girls.^63^ These findings suggested that lower reproductive status was associated with higher total adiponectin concentrations and that a higher reproductive status was related to higher HMW adiponectin concentrations in girls.^63^ In addition, individuals with obesity often develop a chronic low‐grade inflammatory state, which can be indicated by a high level of circulating inflammatory cytokines like TNF‐α and IL‐6.^64^ TNF‐α alters, and IL‐6 inhibits the expression of adiponectin (Figure 2).^8^ Thereby, a low level of total adiponectin and/or high levels of inflammatory cytokines in individuals with obesity can promote the onset of puberty.

Many more adipokines are secreted by WAT including omentin, visfatin, resistin, and chemerin.^52^, ^65^, ^66^, ^67^ In women, omentin, chemerin, and visfatin are expressed in the ovaries.^65^, ^66^, ^67^ The expression of these adipokines in the ovaries suggests a role within the reproductive system; however, the exact biological processes have to be examined. Thus, specifically leptin, adiponectin, and inflammatory cytokines produced by WAT could be permissive key players during an early onset of puberty in girls with obesity.^9^, ^36^, ^62^, ^68^ As an exception, HMW adiponectin seems to have a stimulatory effect on peripheral reproductive function as HMW is not able to cross the blood brain barrier.^63^

## 4 THE ONSET OF PUBERTY IN BOYS

Markers that are used to assess puberty onset in boys are spermarche, voice break, testicular volume, and pubic hair development.^35^ While pubic hair development, larger testicular volume, and spermarche develop in the early stages of puberty onset, voice break usually appears in later stages of puberty.^69^ Generally, first testicular volume increases, which occurs at an average age of 11.9 years, followed by the development of pubic hair at 12.2 years of average, and lastly, boys experience spermarche around an average age of 13.4 years.^70^

### 4.1 Fat storage

Many aspects of the reproductive physiology are energetically demanding,^71^ and therefore, an adequate energy level is necessary. In boys, a dynamic change in body composition occurs around the age of 10 to 13 years, in which they gain approximately 40% of fat.^72^ Subsequently, a growth spurt follows in which they gain tissue mostly consisting of lean mass, which causes exhaustion of most of their body fat.^72^ These alterations in amount of body fat indicate that in boys, an adequate amount of body fat is important in the onset of puberty.^73^

### 4.2 HPG axis

Puberty in boys is initiated by the release of kisspeptin. As mentioned before, this activates the HPG axis, resulting in the release of GnRH from the hypothalamus, and consequently the release of LH and FSH from the pituitary gland (Figure 1).^9^, ^74^ FSH induces spermatogenesis, and LH stimulates the secretion of testosterone from the testes, which inhibits the release of kisspeptin from the KNDy neurons and subsequently GnRH from the hypothalamus.^9^, ^48^ Contrarily to women, in men, the release of kisspeptin is more consistent, causing a constant release of LH.^29^, ^48^ LH‐induced testosterone levels lead to the development of secondary sex characteristics in boys.^9^ In more detail, differences between sexes in kisspeptin release are related to a sex‐specific and sex steroid‐dependent kisspeptin system as estrogen and progesterone modulate kisspeptin activity through the sex‐steroid receptors expressed on KNDy neurons.^48^ In humans, KNDy neurons in the infundibular nucleus are involved in negative and positive sex‐steroid feedbacks.^48^ These sexual dimorphisms are induced by perinatal exposure to sex steroids and result in sex‐specific differences in kisspeptin release.^75^, ^76^

### 4.3 Adipokines

The association between obesity and puberty onset in boys is rather controversial compared with findings in girls. Most studies reported an early onset of puberty in boys associated with increased BMI,^14^, ^17^, ^22^, ^23^, ^50^, ^51^, ^77^, ^78^ while others reported no associations at all^20^, ^49^ or a delayed onset of puberty^79^ (Table 1).^16^, ^80^ The presence of excessive adipose tissue can be involved in puberty onset in boys as the secretion of adipokines can modulate both adrenarche and gonadarche. Leptin can affect adrenarche by modulating both the HPG and HPA axes,^33^ and moreover, androgen levels were positively associated with plasma leptin levels.^55^ Nevertheless, enhanced adrenal androgen secretion in boys with premature adrenarche was not related with leptin levels.^55^ Thereby, leptin plausibly has a minor impact in adrenarche in boys.

**Figure 3:**
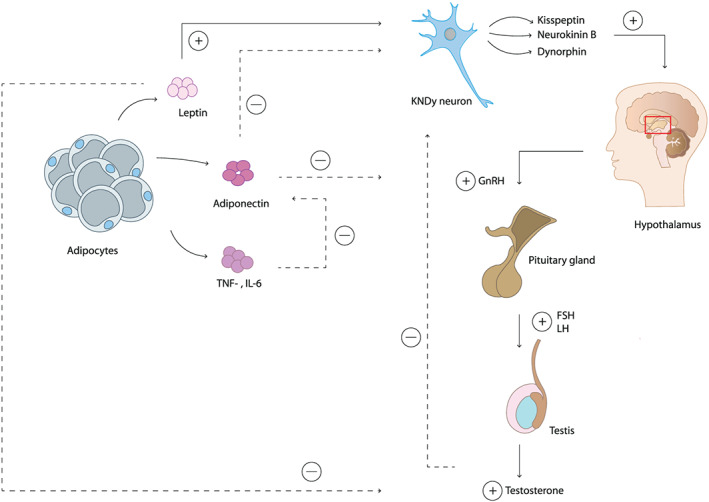
Adipokines affecting the initiation of puberty in boys. Leptin activates kisspeptin secretion in KNDy neurons, this activates the production of gonadotropin releasing hormone (GnRH) from the hypothalamus. GnRH stimulates the pituitary gland to secrete follicle stimulating hormone (FSH) and luteinising hormone (LH), activating the production of testosterone from the testes allowing the development of secondary sex characteristics. Leptin also inhibits the production of testosterone, which may cause a delayed onset of puberty. Adiponectin inhibits GnRH release. Low levels of adiponectin, as a result of TNF‐α and IL‐6 expression, lead to a reduced inhibition of GnRH. In response to GnRH release, the pituitary gland will secrete FSH and LH, and the testes will produce testosterone resulting in the development of secondary sex characteristics in boys

Since leptin receptors are found in the hypothalamus, pituitary gland, and testes, they might be involved in the onset of puberty by affecting the HPG axis during gonadarche. Leptin stimulates the release of kisspeptin and GnRH, and as a consequence, it accelerates the onset of puberty (Table 1, Figure 3). In contrast, adiponectin inhibits the secretion of GnRH, GH, LH, and FSH therewith delaying the onset of puberty. However, adiponectin levels are generally lower in men compared with women and even lower in men with obesity.^61^, ^62^ Moreover, inflammatory cytokines, TNF‐α, and IL‐6, inhibit adiponectin, and individuals with obesity often have high levels of circulating inflammatory cytokines.^64^ High leptin and low adiponectin levels can stimulate the HPG axis and therewith an early onset of puberty in boys. Nevertheless, leptin can inhibit the production of testosterone from the testes,^58^ and fat tissue can convert testosterone to estrogen (Figure 3).^2^, ^60^, ^61^, ^62^ Both processes might result in the delay of the development of secondary sex characteristics in boys.^29^, ^61^, ^79^ Additionally, leptin can affect fertility in men as it can modulate the nutritional support of spermatogenesis, and moreover, dysfunction of spermatogenesis is associated with an increased leptin level and expression of the leptin receptor in the testis.^81^, ^82^

In men, other adipokines like chemerin are found in the gonads too.^65^ Thereby, resistin is expressed in the testes of rats, but its exact function and role still have to be examined.^83^ Thus, particularly high leptin and low adiponectin levels stimulate the HPG axis and thereby accelerate the onset of puberty in boys. Additionally, leptin can dysregulate the development of secondary sex characteristics and spermatogenesis by affecting testosterone levels and nutritional support of spermatogenesis.

## 5 LIMITATIONS AND FUTURE RESEARCH DIRECTIONS

**Figure 4:**
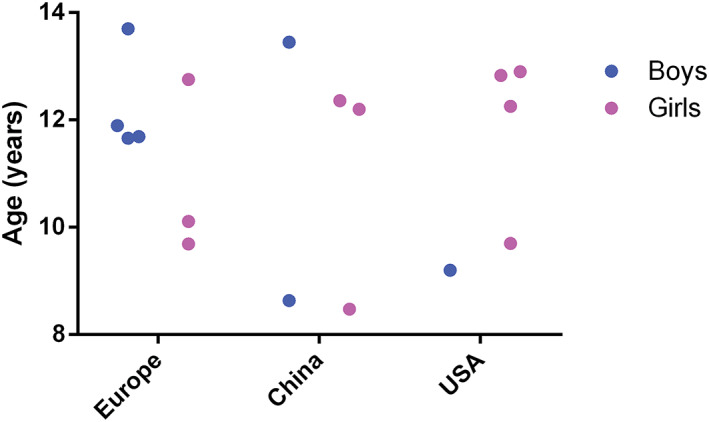
Average age of puberty onset in Europe, China, and the United States according to several studies from Table 1. Age of puberty onset ranges from 8.47 to 13.33 years in girls and from 8.63 to 13.7 years in boys.^12‐15, 17, 20‐23, 25‐29, 31^ Studies (Table 1) were not included if average age of markers used to assess puberty was not reported. Pink: girls. Blue: boys

Even though multiple epidemiological studies have shown the link between puberty onset and obesity, there are some important limitations. Firstly, determining both the onset and stage of puberty is rather difficult. For instance, assessing the stage of breast development in girls with obesity is complicated as clinicians should differentiate adipose tissue from actual breast tissue.^2^ Secondly, male pubertal stages are more difficult to assess than female stages as boys lack a more determined marker such as menarche. Thirdly, puberty onset can be indicated by the activation of the HPG axis, and the presence of these secondary sex characteristics is the result of hormonal changes in response to the activated HPG axis.^2^ Current markers used to determine the onset of puberty refer to secondary sex characteristics, such as testicular volume in boys and breast development in girls. A more accurate measurement of puberty onset would be to combine secondary sex characteristics with plasma or serum hormone level measurements such as LH, FSH, adipokines, e.g. leptin. Thereby, differences in puberty measurements could explain variations in the age of puberty onset between boys and girls within different studies or across continents, countries, and ethnicities (Figure 4).^12^, ^13^, ^14^, ^15^, ^17^, ^20^, ^21^, ^22^, ^23^, ^49^, ^77^, ^78^, ^79^, ^84^, ^85^ In addition, the inclusion of a proper age range (8‐16 years) is important when assessing the onset of puberty.^30^, ^47^ Furthermore, comparison between studies from different time points is complicated, as subjects examined several decades ago presented pronounced differences concerning lifestyle patterns such as nutrition and exercise habits. Lastly, obesity or overweight is often determined by BMI, a classification based on weight and height measurements. Additionally, it is important that all studies use the same anthropometric standards and sex‐specific cut‐offs.^86^ Specifically in children, BMI is often dependent on age and growth spurts.^13^, ^14^, ^16^, ^17^, ^18^, ^19^, ^20^, ^21^, ^22^, ^23^, ^49^, ^50^, ^51^, ^77^, ^78^, ^79^, ^80^ Thereby, BMI is a less accurate measurement in case of growth spurts.^87^, ^88^ Therefore, both percentage and distribution of body fat should be taken into account in determining puberty and obesity in children. For instance, the body adiposity index (BAI), which was introduced in 2011 by Bergman et al., 87 uses hip circumference and height in order to estimate the percentage of body fat and would represent a more accurate measurement in its regard.^87^

Based on this review, several suggestions can be made for further research. Firstly, the roles of adipokines like resistin, chemerin, visfatin, and omentin in puberty onset, fertility, and sexual maturation should be examined in detail. Secondly, future research examining the onset of puberty should combine indicators of puberty onset (e.g. breast development or testicular volume) with plasma or serum hormone measurements such as LH, FSH, sex‐steroids, adipokines (e.g. leptin), and body fat distribution (e.g. BAI,^87^ waist‐hip ratio's and/or dual‐energy X‐ray absorptiometry (DXA)^2^). Additionally, defining consistent and general measurements of puberty in both boys and girls, combined with a proper age range (8‐16 years), would facilitate the comparisons between different studies and their results.^39, 56^

## 6 CONCLUSION

In conclusion, epidemiological data regarding obesity and puberty onset in girls show similar outcomes as adiposity results in the early onset of puberty in girls. The majority of the studies examining boys with obesity indicate an early onset of puberty, while not all reported an earlier onset of puberty. In detail, high leptin, TNF‐α, and IL‐6 levels combined with low adiponectin levels stimulate the activation of the HPG axis in girls and boys with obesity, and thereby an early onset of obesity.^13, 14, 16‐26, 29‐32^ Nevertheless, leptin can inhibit the production of testosterone in boys and subsequently inhibit the development of secondary sex characteristics affecting spermatogenesis.^5, 45, 50, 51^ Furthermore, several receptors for other adipokines, like resistin and omentin, are present in the testes and ovaries suggesting a role in puberty or reproduction; however, their plausible function is still unknown.^58, 71^ We conclude that adipokines may be key regulators in an early onset of puberty in both girls and boys with obesity, specifically by affecting the HPG axis during gonadarche. Future research should focus on assessing puberty onset by measuring consistent puberty markers and determine the percentage of body fat and its distribution and adipokines and hormone serum levels particularly involved in the HPG axis.

**Search strategy**

We searched PubMed for articles published before November 15^th^, 2019 using relevant keywords, including ‘onset of puberty and adiposity/obesity', ‘onset of puberty', ‘children with obesity', ‘adipose tissue', ‘childhood obesity', ‘adiposity', ‘obesity', ‘adipokine(s)', ‘HPG axis', ‘adipokines ovary/ovaries', or ‘adipokines testes', either alone or in combination. Selection criteria used were English language, longitudinal or cross‐sectional studies assessing the onset of puberty, including menarche, thelarche, spermarche, or voice break, combined with high BMI or obesity/adiposity, and articles assessing or reviewing adipokines and its effects on the reproductive system.

## CONFLICTS OF INTEREST

The authors declare no conflict of interest.

## FUNDING INFORMATION

This research was funded by Europees Fonds voor Regionale Ontwikkeling (EFRO), project BriteN 2016.
